# On the Death of Dr. Virgil O. Hardon

**Published:** 1904-04

**Authors:** Bernard Wolff, T. V. Hubbard, W. S. Goldsmith


### ON THE DEATH OF DR. VIRGIL O. HARDON.

### REPORT OF COMMITTEE FROM ATLANTA SOCIETY OF MEDICINE.

It has pleased God to remove from our midst our beloved fellow member, Dr. Virgil O. Hardon.

We bow in humble submission to the decree of Him who doeth all things well, and this knowledge alone mitigates the intensity of sorrow which his loss occasions.

There has been no one in the history of this society who exerted a more profound influence upon it than our departed friend. His counsel and advice were always sought in moulding its policy, and throughout its varied fortunes his interest never flagged. He gave the benefit of his lucid intellect, his sane and temperate judgment and his ripe experience unstintingly, and they were sure to prove inestimable aids in the conduct of the society's affairs. With his death the keystone has dropped from the arch and the gap will be hard to fill. But it will ever remain draped with the mourning of loving memories.

Dr. Hardon loved his profession and the association of his fellow craftsmen, and his love and enthusiasm, combined with mental gifts of a high order and especial adaptability, placed him in an exalted position in the profession of medicine.

His ability as a diagnostician, his erudition in the principles of surgery and his skill as an operator are too well known and appreciated by the profession, as well as by the laity, to require extended comment. His contributions to medical literature were numerous and marked by that thoughtfulness and perspicacity of style which were characteristic of the man. He sought rather to elucidate and simplify subjects of common and practical interest than to invade the troubled sea of hypothesis and fanciful theory. The introduction into the practice of medicine of the alum enema which has been widely adopted and its merits tested at many bedsides, was due to him and was a fact in which he took no little pride.

As a man he was singularly well balanced. The reciprocal qualities of heart and head united in forming a rarely excellent combination. He was loyal to his friends, forgiving of his enemies, without spite or malice. He was ever ready to assist his less fortunate brother willingly and cheerfully. He was a high-principled, high-minded gentlemen, and his qualities of mind and heart endeared him to many, who, in life, were proud to call themselves his friends.

BERNARD WOLFF, M.D.
T. V. HUBBARD, M.D.
W. S. GOLDSMITH, M.D.

